# Identification of two missing genome segments of tulip streak virus

**DOI:** 10.1128/mra.00301-24

**Published:** 2024-07-31

**Authors:** Yutaro Neriya, Hisashi Nishigawa

**Affiliations:** 1 School of Agriculture, Utsunomiya University, Utsunomiya, Tochigi, Japan; Katholieke Universiteit Leuven, Leuven, Belgium

**Keywords:** tulip, *Hareavirales*, *Konkoviridae*, bunyavirus, NSV

## Abstract

Complete sequences of RNA1 and RNA2 of tulip streak virus (TuSV) were already reported, but other segments were not yet. In this study, we reported RNA3 and RNA4 of TuSV, which shared around 69% nucleotide identity with those of closely related virus, suggesting that these are additional RNA segments.

## ANNOUNCEMENT

Tulip streak virus (TuSV) is classified as a species *Olpivirus tulipae*, belonging to the genus *Olpivirus,* family *Konkoviridae*, order *Hareavirales* (formerly *Bunyavirales*) (1). Two genomic segments, RNA1 and RNA2, of TuSV have recently been identified and annotated as an RNA-dependent RNA polymerase (RdRp) and nucleocapsid for RNA1 and RNA2, respectively (2), but no other TuSV segments were reported.

In October 2023, genomic segments of Lactuca big vein associated phlebovirus (LBV), closely related to TuSV, were deposited in GenBank (LBV016 isolate, accession numbers OR610326–9). LBV has not only RNA1 and RNA2, but also RNA3 and RNA4, which have not yet been identified for TuSV. In this study, we found two additional genomic segments in TuSV and identified the complete nucleotide sequence of these segments.

To find additional TuSV segments, sequences homologous to LBV RNA3 and RNA4 were searched using contigs obtained from the same MiSeq (Illumina, CA, USA) analysis data used to determine RNA1 and RNA2 of TuSV from tulip (*Tulipa gesneriana*) in Toyama Prefecture, Japan (2). For the MiSeq analysis, RNeasy Plant Mini kit (Qiagen, Hilden, Germany) was used to extract total RNA from a TuSV-infected tulip flower, Ribo-Zero rRNA removal kit (Plant) (Illumina) was used to eliminate plant ribosomal RNAs, and KAPA Stranded RNA-Seq Library Preparation kit (KAPA Biosystems, MA, USA) was used to prepare MiSeq library according to the manufacturer’s protocol. The paired-end raw reads (301 bp) were cleaned using Trimmomatic (v0.39, with the following parameters: quality 15, min_length 150, and crop 300) (3) and assembled using SPAdes (version 3.9.0, with the “--careful” option) (4). Default parameters were used except where otherwise noted.

From the MiSeq analysis of the tulip sample, 2,000,255 total reads and 4,989 assembled contigs longer than 200 bp were detected. A BLASTn search using the nucleotide sequence of LBV RNA3 and RNA4 revealed that a 1,448-nucleotides (nt) contig (average coverage is 29.79) and a 1,264-nt contig (average coverage is 19.62) showed high similarity to LBV RNA3 and RNA4. To determine the 5′- and 3′-terminal nucleotide sequences, we used a commercial 5′ rapid amplification of cDNA ends (RACE) system (Invitrogen, CA, USA) with specific primers (Table 1). We determined the nucleotide sequence using the SupreDye Cycle Sequencing Kit (M&S Technosystems, Osaka, Japan) and the Applied Biosystems 3500 Genetic Analyzer (Thermo Fisher Scientific, MA, USA). To determine the 3′ end, we used the 5′ RACE system and the viral complementary RNA in the viral replication intermediates. Obtained terminal sequences were assembled with contigs using ATGC version 9.0.1 (Genetyx, Tokyo, Japan), and nucleotide sequence identities were calculated using GENETYX-MAC version 22.0.5 (Genetyx).

**TABLE 1:** Primers used in this study

Primer name	Sequence (5′ to 3′)	Purpose
TuSV3-5ter-GSP1	TACCTTAAGAAATCTAA	Determine the 5'-end of RNA3
TuSV3-5ter-GSP2	TTGAACGGAAGAGAGGACCGTATTG	
TuSV3-3ter-GSP1	AAACACTTAGCATGTT	Determine the 3'-end of RNA3
TuSV3-3ter-GSP2	TCAGCACTCTCGTAAACCAAACCTAT	
TuSV4-5ter-GSP1	ATTCTACTATGTTGAG	Determine the 5'-end of RNA4
TuSV4-5ter-GSP2	CTTTACCAAATGAGCAAGAGGCATAG	
TuSV4-3ter-GSP1	TCATCTATAATGCTAAT	Determine the 3'-end of RNA4
TuSV4-3ter-GSP2	TCATCAAGTGAGCTCTCCAAGATGAC	

**Fig 1:**
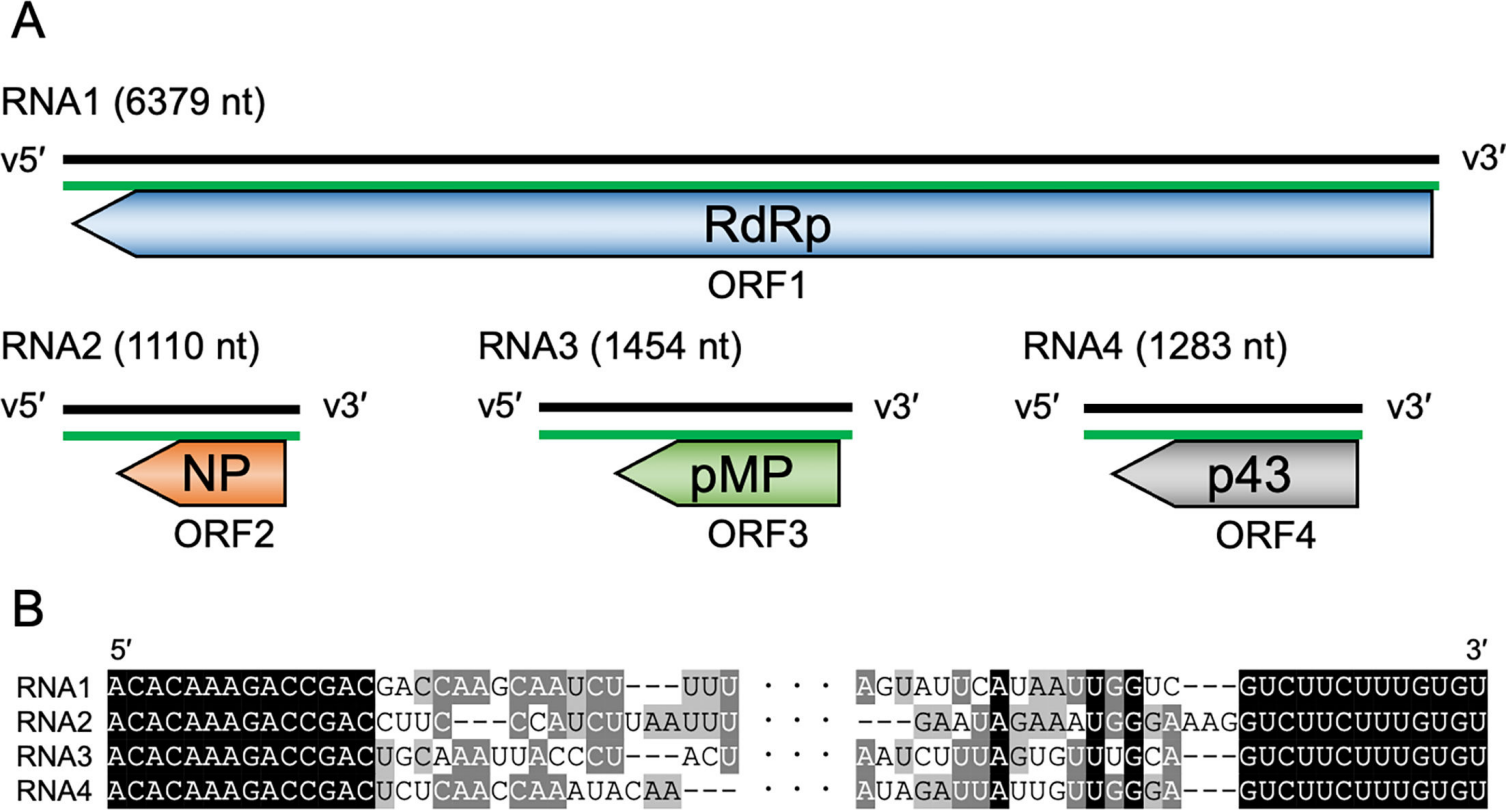
Overview of TuSV genome. (**A**) Schematic overview of viral and complementary strand of TuSV genome. Black and green lines indicate viral RNA and viral complementary RNA, respectively; ORF, open reading frame; RdRp, RNA-dependent RNA polymerase; NP, nucleocapsid protein; pMP, putative movement protein. (**B**) Alignment of the terminal regions (30 nt) of each TuSV genome segment. Black, dark gray, light gray background: bases consensus to 4, 3, and 2 RNA segments, respectively.

We determined the two additional TuSV genome sequences, RNA3 (1,454 nt, 39.4% GC content) and RNA4 (1,283 nt, 39.6% GC content). The length of TuSV RNA3 and RNA4 was 25-nt shorter and 1-nt longer than those of LBV, and the nucleotide sequence identity was 69.3% and 68.8%, respectively. Only one ORF was predicted in each complementary strand (Fig. 1A). The amino acid sequence identities of ORF3 and ORF4 between TuSV and LBV LBV016 isolate were 79.0% and 73.8%, respectively. From the Foldseek (https://search.foldseek.com/search) analysis, ORF3 in RNA3 was predicted to encode a putative movement protein, and the function of ORF4 in RNA4 is unknown.

Like LBV, the sequence of both ends of TuSV RNAs can form a pan-handle structure (2), as shown in Fig. 1B. The terminal nucleotides, 14 nt at the 5′ terminal and 13 nt at the 3′ terminal of each RNA, were conserved and completely identical to those of RNA1 and RNA2 (Fig. 1B). This sequence is also conserved in LBV, suggesting that the terminal sequence is essential for viral replication of these viruses. These results suggested that the two sequences identified were RNA3 and RNA4 of TuSV.

## Data Availability

The genomic nucleotide sequences have been deposited in DDBJ/ENA/GenBank database under the accession number LC805881 (TuSV RNA3) and LC805882 (TuSV RNA4). The MiSeq data set was deposited in the NCBI Sequence Read Archive (SRA) under DRA accession number DRA011074 and SRA run accession number DRR255115.
